# Natural Rubber Latex Foam Reinforced with Micro- and Nanofibrillated Cellulose via Dunlop Method

**DOI:** 10.3390/polym12091959

**Published:** 2020-08-29

**Authors:** Sirilak Phomrak, Adun Nimpaiboon, Bi-min Zhang Newby, Muenduen Phisalaphong

**Affiliations:** 1 Department of Chemical Engineering, Faculty of Engineering, Chulalongkorn University, Phayathai Road, Bangkok 10330, Thailand; loogpoo@hotmail.com; 2 Rubber Technology Research Centre (RTEC), Faculty of Science, Mahidol University, Nakhon Pathom 73170, Thailand; adun.nim@mahidol.ac.th; 3 Department of Chemical, Biomolecular and Corrosion Engineering, The University of Akron, Akron, OH 44325-3906, USA; bimin@uakron.edu

**Keywords:** natural rubber latex foam, Dunlop method, reinforcement, microcellulose, nanocellulose

## Abstract

Natural rubber latex foam (NRLF) was reinforced with micro- and nanofibrillated cellulose at a loading content of 5–20 parts per hundred of rubber (phr) via the Dunlop process. Cellulose powder from eucalyptus pulp and bacterial cellulose (BC) was used as a microcellulose (MC) and nanocellulose (NC) reinforcing agent, respectively. NRLF, NRLF-MC, and NRLF-NC exhibited interconnected macroporous structures with a high porosity and a low-density. The composite foams contained pores with sizes in a range of 10–500 µm. As compared to MC, NC had a better dispersion inside the NRLF matrix and showed a higher adhesion to the NRLF matrix, resulting in a greater reinforcement. The most increased tensile strengths for MC and NC incorporated NRLF were found to be 0.43 MPa (1.4-fold increase) and 0.73 MPa (2.4-fold increase), respectively, by reinforcing NRLF with 5 phr MC and 15 phr NC, whereas the elongation at break was slightly reduced. Compression testing showed that the recovery percentage was improved to 34.9% (1.3-fold increase) by reinforcement with 15 phr NC, whereas no significant improvement in the recovery percentage was observed with MC. Both NRLF-MC and NRLF-NC presented hydrophobic surfaces and good thermal stability up to 300 °C. Due to their highly porous structure, after a prolong immersion in water, NRLF composites had high water uptake abilities. According to their properties, the composite foams could be further modified for use as green absorption or supporting materials.

## 1. Introduction

Cellular rubber, or natural rubber latex foam (NRLF), has long been considered as an economical material with advantageous properties, such as light weight, buoyancy, cushioning performance, thermal and acoustic insulation, inertness, high porosity, robust micro/nanostructures and good aging properties [1,2]. The Dunlop process is a main NRLF manufacturing method invented by E.A. Murphy in the late 1920s for manufacturing latex from the sap of rubber trees. In this process, natural rubber latex (NRL) is compounded with vulcanizing agents and whipped into froth by an assistance of a foaming agent. A delayed-action gelling agent such as sodium silicofluoride (SSF) is added into the fluid foam and subsequently injected into a mold. The fluid foam is allowed to be gelled into solid foam at ambient temperature and baked in a vulcanization oven. After the foam is baked, it is removed from the mold, washed, and then heated a second time to remove moisture from the final product. The result is dense, durable latex foam that retains its elasticity. The Dunlop process is widely used because the production process is simple, reliable, economical and energy-efficient [3]. However, to improve NRLF properties with environmentally friendly construction, natural materials, such as cellulose from plants or byproducts from animals, have been used as reinforcing agents. Bashir et al. [1] attempted to use eggshell to reinforce the NRLF and concluded that the modulus at 100% elongation (M100), compression stress, compression set, hardness, rubber filler interaction and density were increased with an eggshell filler loading up to 10 parts per hundred of rubber (phr). To apply natural fibers, Karim et al. [4] prepared kenaf-filled NRLF and concluded that the increasing content of kenaf reduced the tensile strength, elongation at break and compressive strength of modified NRLF, whereas the M100 and density were enhanced with an increase in filler loading. An agricultural by-product, rice husk powder, was also used as a reinforcing agent for NRLF via the Dunlop process, and it was reported that an increase in rice husk powder loading reduced the elasticity of modified NRLF, resulting in stiffer and more rigid foam, which led to lower resistance to failure (Ramasamy et al. [5]).

In our previous research work, we improved the mechanical and physical properties of natural rubber composite films by reinforcing them with bacterial nanocellulose (BC) via a latex aqueous microdispersion process and found that the tensile strength of NR and BC (NRBC) composite films with BC loaded at 80 wt % was 94% higher than that of pure NR films [6]. BC is a product from primary metabolism processes of microbes, such as *Acetobacter xylinum*. BC has been extensively reported as having unique properties, including high mechanical strength, high water absorption capacity and high crystallinity [6,7,8]. BC is also widely used as biomaterials for medicinal purposes [7,8,9,10,11].

In this study, composites of NRLF reinforced with microcellulose (MC) and nanocellulose (NC) were fabricated via the Dunlop process, in which the pulp of the eucalyptus tree was used as a source of MC and BC was used as a source of NC. The effects of MC and NC loading as reinforcing agents of NRLF on its physical structure, mechanical properties, solvent uptake and thermal stability were investigated. The goal of this work is to develop NRLF composites with improved mechanical properties and absorption capacity, which would be further applied in the biomedical field as wound dressing and as absorption materials for oil–water mixtures.

## 2. Materials and Methods 

### 2.1. Materials

High ammonia NRL (HA-NRL) with 60% dry rubber content (DRC) was purchased from the Rubber Research Institute of Thailand (Bangkok, Thailand). BC (98–99% water content in wet weight) was kindly provided by Pramote Thamarat (Institute of Research and Development of Food Product, Kasetsart University, Bangkok, Thailand). Eucalyptus pulp sheets were kindly supported by the Teppatana Paper Mill Co., Ltd. (Bangkok, Thailand). All other chemical reagents were purchased from Sigma-Aldrich (Bangkok, Thailand).

### 2.2. Preparation of Cellulose Powder

For the preparation of MC, eucalyptus pulp (white cellulose compressed sheets made from 100% cellulose fibers, kindly provided by Teppattana Paper Mill Co., Ltd., Bangkok, Thailand) was soaked in deionized (DI) water for 5 min and was cut into small piece (size 5 × 5 cm^2^). Then it was dried in a general drying oven at 105 °C for 24 h to remove moisture. Next it was grinded into powder by using ball mill (PM 100, Haan, Germany) and sieved in order to obtain the powder with a size of less than 200 µm.

For the preparation of NC, the small cubes of BC hydrogel (size 1 × 1 × 1 cm^3^) were purified by washing with DI water, then soaked with 1 wt % sodium hydroxide (NaOH) for 48 h to remove bacterial cells, and finally rinsed with DI water until the pH was 7.0. After that, BC was thoroughly crushed and homogenized by using a blender at room temperature. Then the BC slurry was dried in a general drying oven at 105 °C for 24 h to remove moisture. Next it was grinded into powder by using ball mill (PM 100, Haan, Germany) and sieved in order to obtain the powder with a size of ≤ 200 µm.

### 2.3. Characterization of Cellulose 

Scanning electron microscopy (SEM) micrographs were taken on a JSM-5410LV (JEOL, Tokyo, Japan). The eucalyptus pulp and BC slurry were dried and sputtered with a 200-Å layer of gold in a Balzers-SCD 040 sputter coater (Liechtenstein). The images of cellulose fibers were immediately viewed at an accelerating voltage of 2 kV.

The definitive structural information and crystallinity were characterized by X-ray diffraction (XRD). XRD patterns of the cellulose powder were determined with a diffractometer (Bruker AXS Model D8 Discover) under 40 kV and 30 mA. X-ray diffraction measurements were performed in the 2θ range of 5–40° using Cu-Ka radiation. The percentage of crystallinity were obtained from the X-ray empirical method proposed by Segal et al. [12].

Thermogravimetric analysis (TGA) of MC and NC powders were performed using a TGA Q50 V6.7 Build 203, Universal V4.5A (TA Instruments, New Castle, DE, USA) equipped with a platinum cell in a nitrogen atmosphere. The scanning range was 35–600 °C using a heating rate of 10 °C min^−1^. The temperature for different percentages of weight loss, the initial degradation temperature (T_d_), and temperature at maximum decomposition were determined from the TGA and differential thermogravimetry (DTG) curves.

### 2.4. Preparation of NRLF and Reinforced NRLF

First, NRL was thoroughly mixed with MC or NC powders dispersed in DI water (at concentration varied from 0, 5, 10, 15 and 20 phr) for 5 min in a beater homogenizer (Kenwood, KVL6320S). Then the mixture was added with potassium oleate soap to make foam until the volume increased up to three times the initial volume (beating time 3 min). After foaming, sulfur, zinc diethyldithiocarbamate (ZDEC), zinc 2-mercaptobenzhiozolate (ZMBT) and phenolic-type antioxidant (Wingstay L) were added to the foam and mixed at low speed for 2 min. Then ZnO slurry and 1,3-diphenylguanidine (DPG) were added as the secondary gelling agent and mixed at low speed for 2 min. Next, the primary gelling agent, sodium silicofluoride (SSF), was quickly added, and the mixture was homogenized for another 90 s. The details on the main compounds and their amounts in the composites are summarized in Table 1. Finally, the foam was immediately poured into the aluminum mold and allowed to be gelled for 3 min at ambient temperature. The gelled NRLF was then vulcanized in a steam oven at 100 °C for 1 h. The vulcanized foam was stripped out from the mold and aged at ambient temperature for 24 h. The aged NRLF was washed with DI water to remove the potassium oleate soap and excessive non-reacted elements. After washing, NRLF was dried in a hot-air oven at 70 °C for 24 h. 

NRLF reinforced with MC in concentrations of 5, 10, 15 and 20 phr are named as NRLF-MC5, NRLF-MC10, NRLF-MC15 and NRLF-MC20, respectively, and NRLF reinforced with NC in concentrations of 5, 10, 15 and 20 phr are named as NRLF-NC5, NRLF-NC10, NRLF-NC15 and NRLF-NC20, respectively. 

### 2.5. Characterization of NRLF, NRLF-MC and NRLF-NC

SEM images were taken with a SEM (model JSM-5410LV, JOEL, Tokyo, Japan). The samples of NRLF, NRLF-MCs and NRLF-NCs were frozen in liquid nitrogen, immediately snapped and then vacuum-dried. The free surfaces were sputtered with gold and imaged. SEM was obtained at 15 kV.

Foam density of NRLF, NRLF-MCs and NRLF-NCs was determined using an analytical balance with a density kit. The report values were the average values determined from five specimens.

Crosslink density of NRLF, NRLF-MCs and NRLF-NCs was determined using swelling method at room temperature according to ASTM D471. Each test specimens were cut into 2 × 2 × 0.6 cm^3^. The weight of each sample was measured before and after immersed into toluene for 48 h. The cross-link density was determined by equilibrium swelling in toluene, based on the Flory–Rehner equation [13,14,15] as shown in Equation (1).

(1) $$-\left\{\ln\left(1-V_{r}\right)+V_{r}+\chi V_{r}^{2}\right\}=\rho V_{0}M_{c}^{-1}V_{r}^{\frac{1}{3}}$$

where *χ* is the interaction parameter between rubber and toluene, which is 0.42, *ρ* is the density of rubber composite (g/cm^3^), *V*_0_ is the molar volume of toluene (cm^3^/mol), *M_c_* is the physical crosslink concentration (mol/cm^3^) and *V_r_* is the volume fraction of the rubber in the swollen sample, which is obtained by Equation (2).

(2) $$V_{r}=\frac{\left(\frac{X_{r}}{\rho_{r}}\right)}{\left(\frac{X_{r}}{\rho_{r}}\right)+\left(\frac{X_{s}}{\rho_{s}}\right)}$$

where *ρ_s_* is the density of toluene (g/cm^3^), *ρ_r_* is the density of rubber (g/cm^3^), *X_r_* is the mass fraction of rubber, which can be obtained using Equation (3), and *X_s_* is the mass fraction of toluene, which is obtained by Equation (4).

(3) $$X_{r}=1-X_{s}$$

(4) $$X_{s}=\frac{m_{s}-m_{d}}{m_{s}}$$

where *m_s_* is mass of swollen sample and *m_d_* is dry mass. After estimating *M_c_* from Equation (1) using the experimentally determined *m_s_* and *m_d_*, the physical crosslink density, *[X]_phys_*, can be obtained by Equation (5) [16].

(5) $${\left[X\right]}_{phys}=\frac{1}{2M_{c}}$$

For tensile property testing, samples of NRLF, NRLF-MCs and NRLF-NCs were cut into a dumbbell shaped specimen, with an overall length of 115 mm and a narrow section of 33 mm wide (according to die C). The maximum tensile strength and break strain of the samples were determined with a universal testing machine (Hounsfield model H10 KM, USA). The test conditions followed ASTM D412. The reported tensile strength and break strain were the average values determined from 5 specimens.

Compression test for NRLF, NRLF-MCs and NRLF-NCs were performed through a Hounsfield H 10 KM universal testing machine (Scientific and Technological Research Equipment Centre, Chulalongkorn University, Bangkok, Thailand). The samples were cut into of 50 × 50 × 25 mm^3^ (length × width × thickness). The samples were placed between the plates of the compression device and compressed to 25% of its original thickness. Within 15 min, compressed specimens and the device were placed in an air oven for a period of 22 h at a test temperature of 70 ± 2 °C. After 22 h, the specimens were immediately removed from the device and allowed to recover for 30 min. Thickness of the specimens was measured after the recovery. Compression properties, such as constant deflection compression set and recovery percentage, were calculated according to ASTM C165. 

The water contact angle under ambient condition was measured by using a contact angle goniometer (Ramé-hart. Instrument Co., USA, model 100-00) equipped with a Gilmont syringe and 22-gauge flat-tipped needle.

The water uptake of NRLF, NRLF-MCs and NRLF-NCs was determined at room temperature according to ASTM D471. Each test specimens were in the form of 2 × 2 × 0.6 cm^3^. The mass of each sample was measured before and after immersed into water for 72 h. The degree of uptake was calculated as;

(6) $$Degree\;of\;uptake\;\left(\%\right)\;=\frac{W_{w}-W_{d}}{W_{d}}\times 100$$

where, *W_w_* and *W_d_* denote the weight of wet and dry samples, respectively.

Thermal stability of the composite foams was performed using a TGA Q50 V6.7 Build 203, Universal V4.5A (TA Instruments, New Castle, DE, USA) equipped with a platinum cell in a nitrogen atmosphere. The scanning range was 35–600 °C using a heating rate of 10 °C min^−1^. The temperature for different percentages of weight loss, T_d_, temperature at maximum decomposition, and residue level at 600 °C of NRLF and NRLF-MCs and NRLF-NCs were determined from the thermogravimetric analysis (TGA) and derivative thermogravimetric (DTG) curves. 

## 3. Results and Discussion

### 3.1. Characterization of Cellulose Fibers/Powders

**Figure 1:**
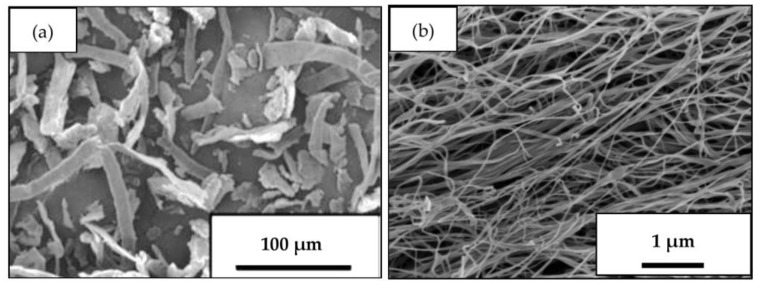
SEM images of microcellulose fibers from eucalyptus pulp (**a**) and nanocellulose fibers from BC (**b**).

According to the SEM results, the average width and length of dry fibers were around 10 and 100 µm, respectively for the eucalyptus pulp (Figure 1a) and 10 and 3000 nm, respectively for BC (Figure 1b). The MC and NC powders were fiber packed particles in the size around 0.5–200 µm. Small portion of MC particles with a size smaller than 1 µm was detected, which indicated that some of the pulp fibers were pulverized into smaller particles during mechanical grinding in a ball mill [17,18]. The particle size of NC powder was considerably larger than the diameter of bacterial nanocellulose (BC) fiber, which could be due to the agglomeration of BC fibers during the drying process. The occurrence of this phenomenon was probably the result of the large specific area and strong hydrogen bonding between the particles, which were not affected by a mechanical grinding method [19].

**Figure 2:**
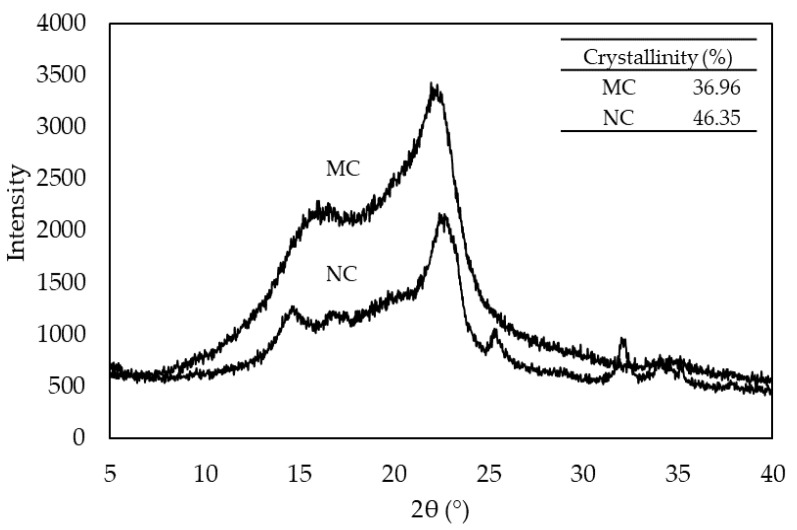
XRD patterns of microcellulose (MC) and nanocellulose (NC) powders.

XRD patterns of MC and NC powders are shown in Figure 2. Three main peaks of MC were detected at 2θ = 14.9°, 16.5° and 22.6°; however, it was difficult to observe a peak at 14.9° from the MC diffractograms because of two overlapping peaks between the first peak (14.9°) and the second peak (16.5°). NC showed peaks at 14.1°, 16.1° and 22.4°. Those peaks correspond to $1\bar{1}$0, 110 and 020 lattice planes of the cellulose I crystalline form [20,21]. The locations of peaks were similar to those of plant celluloses [15,16] and BC [17,18,19]; however, their diffraction peaks were relatively less sharp. Therefore, the mechanical grinding process by using a ball mill might partially reduce the crystalline regions, leading to a higher proportion of the amorphous phase. MC showed less sharp peaks compared to those of NC, indicating a less crystalline material. The crystallinity of MC and NC powders were 36.96% and 46.35%, respectively. The XRD diffractogram of NC also showed peaks at 28–40°, which could be connected with NaOH presence in NC [22], because NaOH solution was used as the solvent to remove bacterial cells from NC. Cellulose treated with NaOH could react with this substance, which caused the incorporation of sodium into the cellulose structure [23], the most probably in ionic form [24,25]. Crystalline NaOH have diffraction patterns at another 2θ value [22] than this observed result. The most intensive patterns for NaOH have to be at about 38 and then 32° [26]. In this case the observed patterns could be most probably connected with Na-cellulose formation [24].

### 3.2. Morphology of NRLF, NRLF-MC, and NRLF-NC

**Figure 3:**
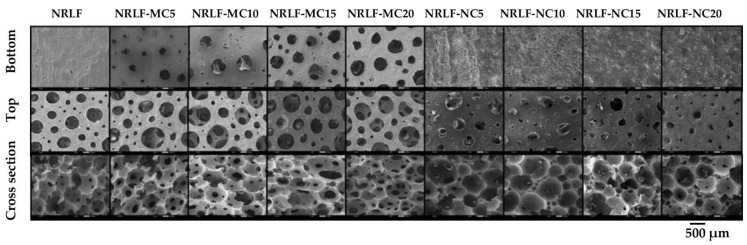
SEM images of surfaces (bottom and top) and cross section views of NRLF composites.

Previous studies have reported reinforced NRLF with eggshell powder [1], kenaf powder [4] and rice husk powder [5] with loading up to 10 wt %. From our preliminary study, it was found that adding fibers from plant cellulose or BC in the form of dry or wet fibers was also limited at about 10 wt % due to poor dispersion of cellulose in the NR matrix. In addition, at a high loading of cellulose fibers, the occurrence of fiber bloom on the surface was observed. To enhance the cellulose loading content, MC and NC were, therefore, prepared in the form of powder, and then were added with DI water to form dense slurry. The optimal weight ratio for MC slurry was MC:DI water at 50:50; the optimal weight ratio for NC slurry was NC:DI water at 75:25. Well-dispersion of cellulose particles in the NR could be obtained by thoroughly mixing the dense slurry with the NR. By this modified method, a higher content of cellulose loading, up to 20 phr, could be applied for the fabrication of NRLF-MC and NRLF-NC. The structures of NRLF, NRLF-MCs and NRLF-NCs, imaged by SEM are shown in Figure 3. The cross-sectional views of NRLF composites at MC and NC loading from 5 to 20 phr showed that MC and NC were well dispersed within the NR matrix without segregation between cellulose particles and NR.

Observation of the porous structure showed that no pores were on the bottom surface of the NRLF; however, numerous pores in the range of 10–400 µm were observed on the top surface and in the cross-sectional view. NRLF exhibited an interconnected macroporous structure of high porosity. Commonly, the Dunlop method is a mechanical foaming process that regulates pore formation poorly, leading to a non-homogeneous foam distribution. NRLF-MC exhibited rather similar structure in the cross-sectional view and on the top surface compared with NRLF; however, the pore size was relatively larger. Open macropores were also observed on the bottom surface of NRLF-MC and the number of pores increased with the loading content of MC. NRLF-NC presented a foam structure with no pores on the bottom surface and fewer and smaller pores were observed on the top surface as compared to those of NRLF and NRLF-MCs. All cross-sectional views of NRLF-MC and NRLF-NC showed an interconnected macroporous structure with pores sizes in the range of 10–500 µm. It demonstrated that reinforcement with cellulose fillers changed the porous structure of NRLF composites. The size and loading content of cellulose fibers were important factors affecting the porous structure of NRLF composite foams fabricated with the Dunlop method. 

### 3.3. Foam Density

**Figure 4:**
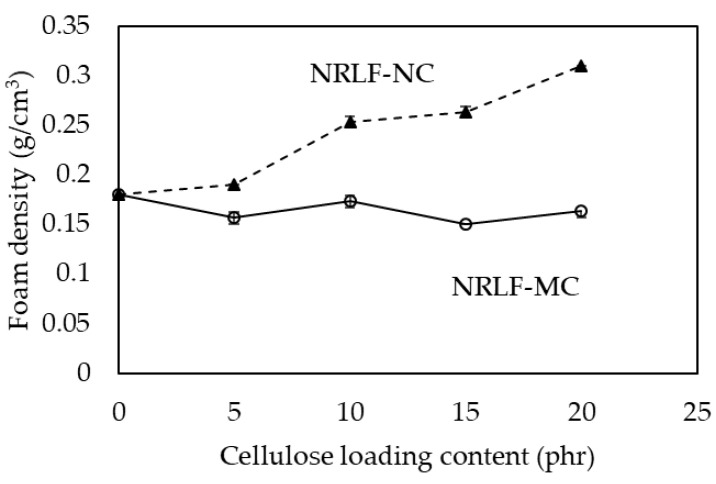
Foam densities of NRLF, NRLF-MC and NRLF-NC.

Densities of NRLF, NRLF-MCs and NRLF-NCs are shown in Figure 4. In general, the density is enhanced with the addition of filler, if the total volume of composites remains constant. There was a slight change in the foam density of NRLF-MC by MC loading; the foam density was 0.15–0.18 g/cm^3^, where the density of NRLF was 0.18 g/cm^3^, which should be due to the similar pore structure of NRLF and NRLF-MC. The increase in MC loading, along with the increase in pore size and pore volume, caused no dramatic change in NFLC-MC density as compared with that of NRLF. However, NRLF-NC demonstrated fewer and smaller pores on the top surface as compared with NRLF, and the density of NRLF-NC considerably increased with NC loading content, as shown in Figure 4. The highest density of 0.31 g/cm^3^ (about 1.7 times of that of NRLF) was obtained by NC loading at 20 phr.

### 3.4. Crosslinking Density

**Figure 5:**
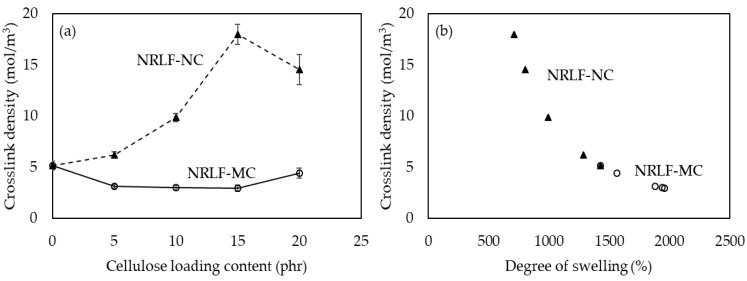
Crosslink density as a function of (**a**) cellulose loading content and (**b**) degree of swelling in toluene. The filled triangles and open circles are for NRLF-NCs and NRLF-MCs, respectively.

The crosslinking density of the vulcanized rubber determines their mechanical properties in which the increase in the crosslink density lead to the improvement of mechanical properties [27,28]. It was determined by equilibrium swelling in toluene and based on the Flory–Rehner equation [13,14,15]. The results are shown in Figure 5. According to Figure 5a, the crosslinking density of NRLF-NC was increased with an increase in loading content of NC particles, which should be due to an increase in interaction between NC fibers and NR and hydrogen bonding between NC fibers in NR matrix, making it harder for the composite to expand in toluene. In addition, the polarity and tortuosity might be responsible for the restricted penetration of toluene into the NR matrix [29]. As compared to NRLF-NC 15, the crosslink density of NRLF-NC20 was lower, which could be due to less uniform dispersion of NC particles in the NR matrix. 

A higher foam density would normally lead to a higher crosslinking density [30]. In case of NRLF-MCs, relatively lower crosslink densities were observed when compared to NRLF-NCs, which might be owing to their lower foam densities. Generally, a higher crosslink density presents a lower solvent swelling ability, because the increase in crosslink density hinders polymeric chain mobility [31]. According to Figure 5b, NRLF-MCs and NRLF-NCs followed the expected trend, in which the increase in degree of swelling is related to the decrease in crosslinking density. NRLF-NCs showed lower degrees of swelling when compared to NRLF-MCs.

### 3.5. Mechanical Properties

Generally, modulus and tensile strength would be improved with an increase in loading content of reinforcing fillers. The smaller fillers and better distribution/dispersion of fillers in composite matrix are also favored for mechanical enhancement [32]. It was reported that fillers with smaller size, specifically at the nanoscale, showed a better efficiency in improving modulus and tensile strength than those with a larger size, i.e., at the microscale [33,34]. Good adhesion between fillers and the matrix, which promotes the efficiency of reinforcement, is also important for improving mechanical properties. In contrast, low interaction, which could cause agglomeration of reinforcing particles, consequently low dispersion of reinforcing particle in the main polymer matrix, hindering the efficiency of reinforcement [35].

**Figure 6:**
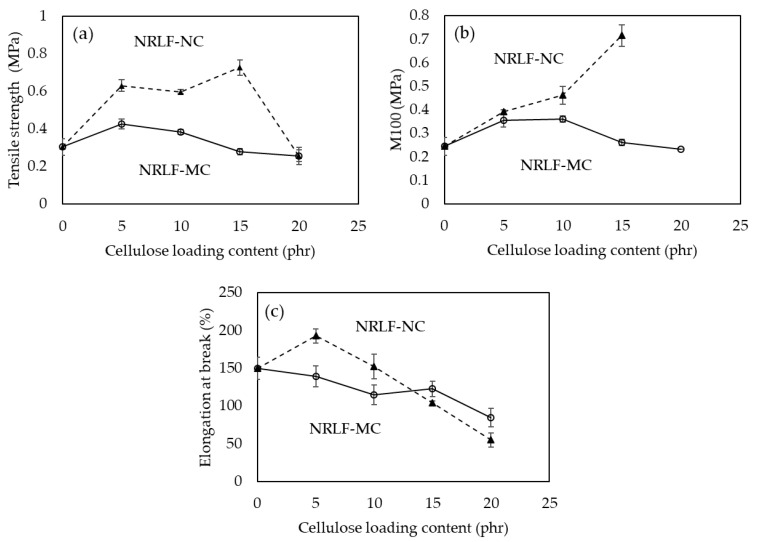
Mechanical properties: (**a**) tensile strength, (**b**) M100 and (**c**) elongation at break of NRLF, NRLF-MC and NRLF-NC.

The tensile strength of NRLF-NC increased after reinforcement with 5, 10 and 15 phr of NC, and NRLF-NC15 showed the highest value of tensile strength at 0.73 MPa (Figure 6a). However, NRLF-NC20 showed reduced tensile strength of 0.26 MPa. The result indicated, as compared to 20 phr, better dispersion occurs with NC loading at 5, 10 and 15 phr, likely due to better interactions between NC particles and the NR matrix. Good interaction and well dispersion of nanocellulose within the NR matrix lead to efficient stress transfer from the matrix to nanocellulose particles [36]. Nonetheless, a reduced effect was observed when reinforcing NRLF with NC at 20 phr, which was probably caused by non-uniform dispersion of NC particles in the NR matrix. Under this limitation, the M100 (modulus at 100% elongation) of NRLF-NC20 could not be determined, whereas NRLF-NC5, NRLF-NC10 and NRLF-NC15 showed an increased in M100 as compared with that of NRLF, and the value of M100 increased with the increased NC loading from 5 to 15 phr (Figure 6b). The crosslink density has an effect on modulus. M100 has been reported to increase with the increase in crosslink density of vulcanized rubber [27]. The network structure formed under crosslinking conditions is a key to increase the modulus and strength of cross-linked rubber composites [37]. The elongation at break was also improved for NRLF-NC5, but it was reduced by reinforcing NRLF with NC at greater than 5 phr (Figure 6c). Since NC particles dispersed in a NR matrix could immobilize NR chains, thus hindering the molecular mobility of NR, which could result in reduced elasticity and an increased stiffening phase from NC loading [38].

In case of NRLF-MCs, the tensile strength was increased to 0.43 MPa after reinforcing with MC at 5 phr, and the tensile strength gradually decreased with the addition of MC from 10 to 20 phr. The result might be due to the increase of pore size and the decrease in foam density of NRLF-MC as compared with NRLF. MC loading had an effect on the M100 value of NRLF-MC, similar to that on the tensile strength. The elongation at break of the composites was also found to be decreased with an increase in MC loading.

To compare reinforcing efficiency between MC and NC, the highest values of tensile strength and M100 were obtained by reinforcing NRLF with NC at 15 phr, which were 1.7 and 2.0 times, respectively, the maximum values obtained from reinforcing NRLF with MC. The results demonstrated that the smaller size of the NC fillers could be dispersed more thoroughly into the NR matrix than the larger MC fillers, promoting more interactions between the filler and NR. The higher crystallinity of NC could also enhance NRLF-NC strength. When crystalline NC was added into the amorphous matrix of NR, the crystallinity of the composite probably dominated the bulk properties, resulting in increased modulus values [39,40,41]. Meanwhile, the increase in pore size in the NR matrix via MC loading could lead to a reduction in tensile strength.

### 3.6. Compression Properties

**Figure 7:**
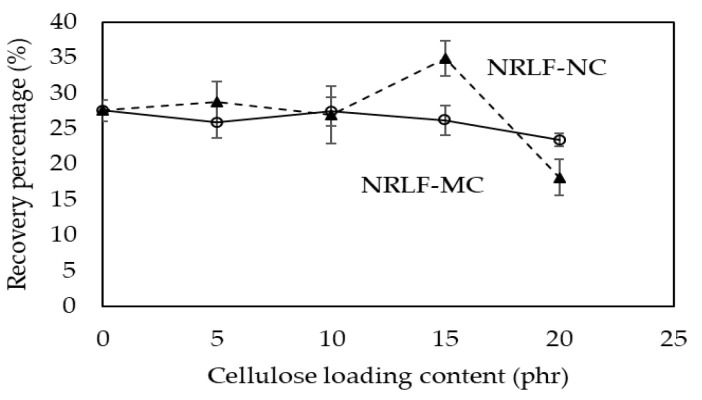
Recovery (%) from the compression test of NRLF, NRLF-MC and NRLF-NC.

Compression tests were performed to evaluate the composite elasticity. In these tests, samples were placed between the plates of the compression device and compressed to 25% of their original thickness. In general, the elasticity in terms of recovery percentage was decreased with increased reinforcing particle loading, which is the stiffening phase. As shown in Figure 7, there was no dramatic decline in recovery percentage with increased MC loading in NRLF-MC. Therefore, not only an increase in the hard phase but also the pore structure should have a significant contribution to their elasticity. The increased pore diameters in the NR matrix to some extent decreases the foam stiffness, which could promote the elastic property or recovery percentage [42]. No significant improvement in recovery percentage was obtained by reinforcing NRLF with NC, except for NRLF-NC15. NRLF-NC15 showed the highest recovery percentage at 34.9% (increased by 26.1% with respect to NRLF). As described in Section 3.5, the maximum mechanical properties were obtained by reinforcing NRLF with NC at 15 phr. Therefore, this result supported that the good dispersion of 15 phr NC in the NR matrix could enhance adhesion between NC and the NR matrix, resulting in the improvement of many mechanical properties of NRLF, including compression recovery.

### 3.7. Surface Wettability

**Figure 8:**
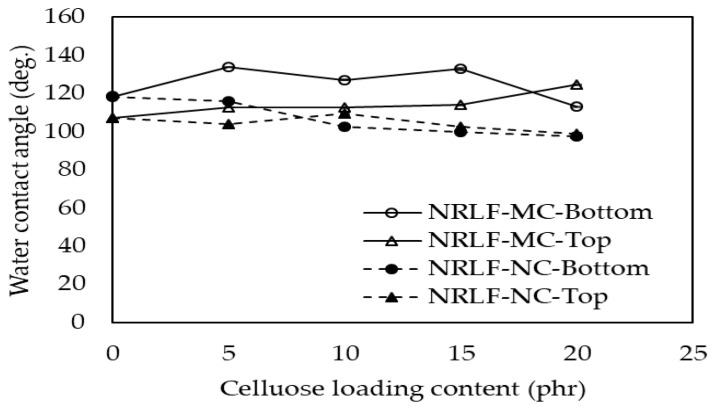
Water contact angle on the bottom and top surfaces of NRLF, NRLF-MC and NRLF-NC.

The water contact angle was used to examine the changes in surface wettability of NRLF, NRLF-MCs and NRLF-NCs. Cellulose, which is rich in hydroxyl groups, is highly hydrophilic, whereas NR is a hydrophobic polymer. As shown in Figure 8, the bottom and top surface of NRLF, NRLF-MC and NRLF-NC showed hydrophobic characteristics. The SEM images in Figure 3 allowed the observation of surface morphology of the samples and it was shown that the roughness of top surface was higher as compared to the bottom surface. For a non-porous NR film, a water contact angle between 90° and 100° has been reported [43] under ambient conditions. If the water contact angle was higher than 90°, the roughness would lead to an increase in water contact angle [44]. Another possibility of these contact angles being greater than the non-porous NR film could be due to trapping of air inside the pores. The more air is being trapped (i.e., occupying more surface area), the greater contact angle would be based on the Cassie–Baxter’s law [45]. Hence, the top surfaces, having a higher roughness and/or containing more and larger pores to trap air, would be expected to show higher contact angles. However, the static water contact angles of the bottom surface of NRLF-MCs were higher than those of the top surface. This result could imply that there was relatively higher density of MC on the top surface, likely due to fiber blooming, than that on the bottom surface. On the other hand, there was no dramatic difference in surface wettability between the top and bottom surfaces of NRLF and NRLF-NCs. The water contact angles were slightly decreased with increased NC loading, which could be due to the increase of OH groups in BC on the surface of the NRLF-NCs. In comparison with NRLF-MCs, NRLF-NCs showed less hydrophobic characteristic, which should be due to the better integration of NC in the NR matrix, as compared with that of MC.

### 3.8. Water Uptake

**Figure 9:**
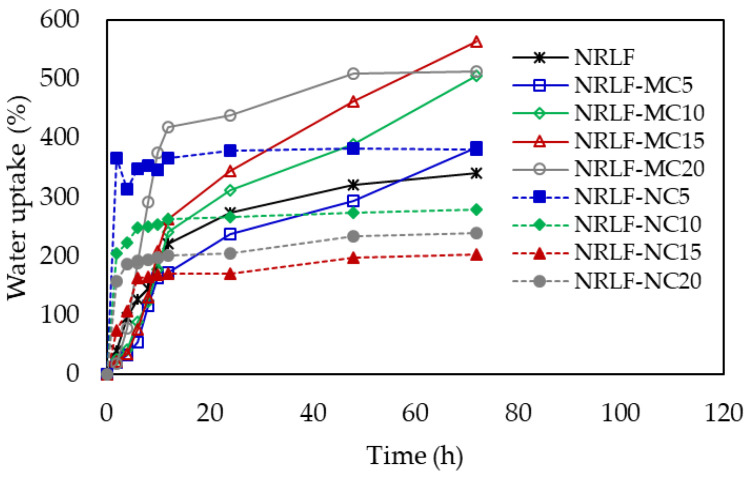
Water uptakes of NRLF, NRLF-MC and NRLF-NC.

Water was used as a polar solvent for the determination of the degree of solvent uptake of NRLF, NRLF-MCs and NRLF-NCs, as shown in Figure 9. For hydrophobic foams, water uptake was not expected. However, we did notice water uptake after the composite foams were immersed in water, potentially due to migration/segregation of hydrophilic constitutes in the foams to the surface. The details on the mechanism were not investigated in this study. NRLF showed high-water uptake rates initially (first 12 h) and then the degree of uptake rate gradually decreased with time. The water uptake of NRLF-MC continuously increased during the 72-h immersion experiment, demonstrating that the absorption might not be saturated at 72 h. At 72 h of absorption, the water uptake capacity of NRLF-MC was higher than that of NRLF; the maximum water uptakes at 513–565% (1.50–1.66 folds of NRLF) were obtained from NRLF-MC10, NRLF-MC15 and NRLF-MC20. The increased water uptake capability of NRLF-MCs should be due to the more porous structure of the NR matrix loaded with MC. NRLF-NC had a water uptake rate pattern similar to that of NRLF; however, a higher water uptake rate was observed at the initial period (6 h), and after that the degree of uptake rate gradually decreased (or the water uptake value remained almost constant) over time. The NRLF-NC composite was saturated with water during 24–72 h immersion. The addition of NC filler increased the water absorption rate initially, but because NRLF-NC composites had a denser structure (less porosity), the maximum water uptakes of NRLF-NC10, NRLF-NC15 and NRLF-NC20 were less than that of NRLF. Only NRLF-NC5 showed slightly higher water uptake capacity than NRLF. The higher porosity of NRLF-MCs could promote the water uptake ability. General, porosity and pore size have effects on the penetration of liquid into porous materials [46], in which higher porosity could promote the liquid uptake capacity.

### 3.9. Thermal Degradation

**Figure 10:**
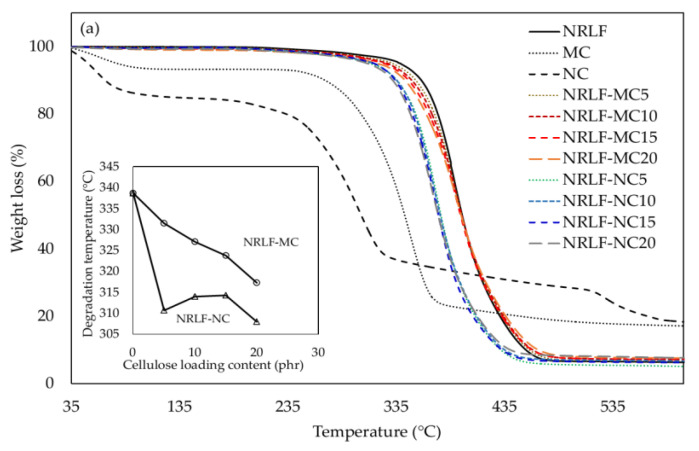

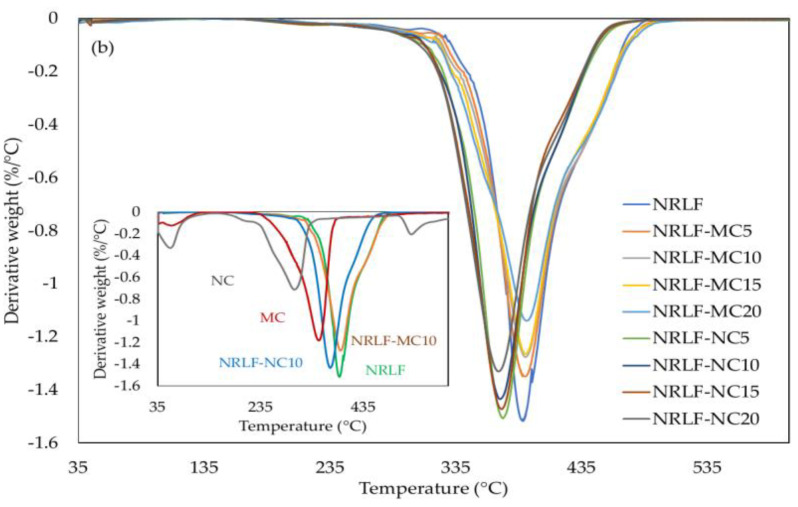
TGA (**a**) and DTG (**b**) curves for the degradation of NRLF, MC, NC, NRLF-MC and NRLF-NC.

The result of thermal degradation is shown in Figure 10. For MC and NC, the peak around 59 °C was for the evaporation of water and the thermal degradation temperatures (T_d_) of NC and MC were 230 and 276 °C, respectively. MC exhibited greater T_d_ than NC, because the degree of polymerization (DP) and the particles size could have influenced on the thermal stability of cellulose. The thermal degradation of NC prepared from BC could be divided into 2 steps. The first step was the main decomposition step, occurring from 200 to 400 °C, where depolymerization, dehydration and decomposition of the cyclic structures were observed [6,47]. The second weight loss of NC from BC corresponded to the pyrolysis of cellulose [47], which was observed from around 528 °C [6,47]. However, this pattern was not observed from MC. The weight loss of MC was observed around 300–400 °C [48,49,50]. It was demonstrated that particle size and crystallinity of cellulose affected the pyrolysis of cellulose [49,50]. According to literature data, the NRLF degradation process included chain scission chain scission, cross-link formation and cross-link breakage [51]. The T_d_ of NRLF was 338.7 °C. The TGA curves revealed a single-step degradation process for all NRLF, NRLF-MC and NRLF-NC specimens, in which the thermal decomposition periods were around 300–500°C. There was no separate degradation step for NRLF-MCs and NRLF-NCs, between NR and MC/NC on DTG pattern (Figure 10b). The result indicates that both NRLF-MC and NRLF-NC exhibited good dispersion and distribution of cellulose particles in the NR matrix with good adhesion between cellulose particles and the NR matrix at a MC/NC loading content from 5 to 20 phr. According to the result, NRLF and the composite foams NRLF-MC and NRLF-NC can be used for applications at temperature up to approximately 300 °C. Previously, it was demonstrated that NR presented a higher thermal resistance than cellulose; T_d_ of NR was higher than that of cellulose [6]. The T_d_ values of NRLF-MC and NRLF-NC were slightly lower when compared with NRLF and tended to decrease with increased cellulose loading, following the rule of mixture, in which T_d_ of composites varied between the values of polymer matrix and fillers. The thermal resistance property was reported for MC and NC used as reinforcing agents [52]. In this study, only NRBC-NC20 and NRBC-NC15 showed significantly lower thermal degradation lines separated from those of the NRLF and the NRLF composites, which should be due to a high concentration of NC dispersed in the NR matrix. 

## 4. Conclusions

NRLF was reinforced with MC and NC via the Dunlop process. According to the SEM results, the average width and length of dry fibers were around 10 and 100 µm, respectively for the eucalyptus pulp, and 10 and 3000 nm, respectively for BC. The crystallinity of MC and NC was 37.0% and 46.4%, respectively. MC and NC fillers at loading contents from 5 to 20 phr (dry basis) could be well dispersed within the NR matrix. The resulting NRLF, NRLF-MC and NRLF-NC were highly porous with interconnected macropores and low-density foams. The pore sizes ranged from 10 to 500 µm. It was demonstrated that the size, crystallinity and loading content of cellulose fibers were important factors affecting the porous structure and properties of NRLF composite foams. On the surface, NRLF-MC presented a more open pore structure, whereas NRLF-NC contained fewer and smaller pores. The foam density and crosslinking density of NRLF-NCs were higher than those of NRLF-MCs and the crosslinking density increased with NC filler loading from 5 to 15 phr. NC also showed reinforcement efficiency higher than that of MC. NRLF-NC, particularly NRLF-NC15, demonstrated significantly improved mechanical properties in terms of tensile strength, M100 and compression recovery. Both NRLF-MC and NRLF-NC presented a hydrophobic surface; however, NRLF-NC showed slightly less hydrophobic characteristics, which likely was the result of a better dispersion of NC particles, as compared to MC particles, within the NR matrix. After a prolonged period of immersion in water, all NRLF composite foams showed a high water uptake; and due to its greater porosity, NRLF-MC had a higher water uptake capacity than those of NRLF and NRLF-NCs. All NRLF composites had good thermal stability and could be used for applications at temperature up to 300 °C. There was no separate degradation step for NRLF-MCs and NRLF-NCs, between NR and MC/NC on DTG pattern. The T_d_ values of NRLF-MC and NRLF-NC were slightly lower than NRLF and tended to decrease with increased cellulose loading. According to their properties, the composite foams could be further modified for use as green absorption or supporting materials. To the best of our knowledge, this is the first report of significant improvement in mechanical properties of reinforcing NRLF foam by nanobacterial cellulose.

## Figures and Tables

**Table 1:** Formulation of the natural rubber latex (NRL) compounds for the synthesis of reinforcing NRL foam (NRLF).

Ingredient	Amount (phr)
60% DRC HA-NRL	100
MC or NC	5, 10, 15, 20
10 wt % Potassium oleate soap	1.5
50 wt % Sulfur	2.0
50 wt % Phenolic Adhesive Antioxidant	1.0
50 wt % ZMBT	1.0
50 wt % ZDEC	1.0
50 wt % ZnO	5.0
33 wt % DPG	1.0
12.5 wt % SSF	1.0

## References

[1] Bashir A.S., Munusamy Y., Chew T.L., Ismail H., Ramasamy S. (2015). Mechanical, thermal, and morphological properties of (eggshell powder)-filled natural rubber latex foam. J. Vinyl Addit. Technol..

[2] Zou L., Phule A.D., Sun Y., Zhu T.Y., Wen S., Zhang Z. (2020). Superhydrophobic and superoleophilic polyethylene aerogel coated natural rubber latex foam for oil-water separation application. Polym. Test..

[3] Rathnayake W.G.I.U., Ismail H., Baharin A., Bandara C.D., Rajapakse S. (2013). Enhancement of the antibacterial activity of natural rubber latex foam by the incorporation of zinc oxide nanoparticles. J. Appl. Polym. Sci..

[4] Karim A.F.A., Ismail H., Ariff Z.M. (2016). Properties and characterization of Kenaf-Filled natural rubber latex foam. Bioresources.

[5] Ramasamy S., Ismail H., Munusamy Y. (2012). Tensile and morphological properties of rice husk powder filled natural rubber latex foam. Polym. Technol. Eng..

[6] Phomrak S., Phisalaphong M. (2017). Reinforcement of natural rubber with bacterial cellulose via a latex aqueous Microdispersion process. J. Nanomater..

[7] Ciechanska D. (2004). Multifunctional bacterial cellulose/chitosan composite materials for medical applications. Fibres Text. East. Eur..

[8] Czaja W., Krystynowicz A., Bielecki S., Brown R.M. (2006). Microbial cellulose—The natural power to heal wounds. Biomaterials.

[9] Deng C.-M., He L.-Z., Zhao M., Yang D., Liu Y. (2007). Biological properties of the chitosan-gelatin sponge wound dressing. Carbohydr. Polym..

[10] Bodhibukkana C., Srichana T., Kaewnopparat S., Tangthong N., Bouking P., Martin G.P., Suedee R. (2006). Composite membrane of bacterially-derived cellulose and molecularly imprinted polymer for use as a transdermal enantioselective controlled-release system of racemic propranolol. J. Control. Release.

[11] Klemm D., Schumann D., Udhardt U., Marsch S. (2001). Bacterial synthesized cellulose—Artificial blood vessels for microsurgery. Prog. Polym. Sci..

[12] Segal L., Creely J., Martin A., Conrad C. (1959). An empirical method for estimating the degree of crystallinity of native cellulose using the X-ray diffractometer. Text. Res. J..

[13] Treloar L.R.G. (1975). The Physics of Rubber Elasticity.

[14] Najib N., Ariff Z.M., Bakar A., Sipaut C. (2011). Correlation between the acoustic and dynamic mechanical properties of natural rubber foam: Effect of foaming temperature. Mater. Des..

[15] Ariff Z., Zakaria Z., Tay L., Lee S. (2008). Effect of foaming temperature and rubber grades on properties of natural rubber foams. J. Appl. Polym. Sci..

[16] Abd-El-Messieh S., El-Nashar D., Khafagi M. (2004). Compatibility investigation of microwave irradiated acrylonitrile butadiene/ethylene propylene diene rubber blends. Polym. Technol. Eng..

[17] Zheng Y., Fu Z., Li D., Wu M. (2018). Effects of ball milling processes on the microstructure and rheological properties of microcrystalline cellulose as a sustainable polymer additive. Materials.

[18] Gao C., Xiao W., Ji G., Zhang Y., Cao Y., Han L. (2017). Regularity and mechanism of wheat straw properties change in ball milling process at cellular scale. Bioresour. Technol..

[19] Zhou L., He H., Li M.-C., Song K., Cheng H., Wu Q. (2016). Morphological influence of cellulose nanoparticles (CNs) from cottonseed hulls on rheological properties of polyvinyl alcohol/CN suspensions. Carbohydr. Polym..

[20] Ling Z., Edwards J.V., Guo Z., Prevost N.T., Nam S., Wu Q., French A.D., Xu F. (2019). Structural variations of cotton cellulose nanocrystals from deep eutectic solvent treatment: Micro and nano scale. Cellulose.

[21] Phisalaphong M., Suwanmajo T., Sangtherapitikul P. (2008). Novel nanoporous membranes from regenerated bacterial cellulose. J. Appl. Polym. Sci..

[22] Liu M., Wang H., Han J., Niu Y. (2012). Enhanced hydrogenolysis conversion of cellulose to C2–C3 polyols via alkaline pretreatment. Carbohydr. Polym..

[23] Nomura S., Kugo Y., Erata T. (2020). 13C NMR and XRD studies on the enhancement of cellulose II crystallinity with low concentration NaOH post-treatments. Cellulose.

[24] Keshk S.M.A.S., Hamdy M.S. (2019). Preparation and physicochemical characterization of zinc oxide/sodium cellulose composite for food packaging. Turk. J. Chem..

[25] Williams T., Hosur M., Theodore M., Netravali A., Rangari V., Jeelani S. (2011). Time effects on morphology and bonding ability in mercerized natural fibers for composite reinforcement. Int. J. Polym. Sci..

[26] Li Y., Yu H.-y., Zhang Z.-t., Zhang M., Guo M. (2015). Selective phase transformation behavior of titanium-bearing electric furnace molten slag during the molten NaOH treatment process. ISIJ Int..

[27] Choi S.-S., Kim E. (2015). A novel system for measurement of types and densities of sulfur crosslinks of a filled rubber vulcanizate. Polym. Test..

[28] Roy K., Debnath S.C., Tzounis L., Pongwisuthiruchte A., Potiyaraj P. (2020). Effect of various surface treatments on the performance of jute fibers filled natural rubber (NR) composites. Polymers.

[29] Dominic CD M., Joseph R., Begum P., Joseph M., Padmanabhan D., Morris L.A., Kumar A.S., Formela K. (2020). Cellulose nanofibers isolated from the cuscuta reflexa plant as a green reinforcement of natural rubber. Polymers.

[30] Khimi S., Syamsinar S., Najwa T. (2019). Effect of carbon black on self-healing efficiency of natural rubber. Mater. Today Proc..

[31] Zheng L., Li C., Zhang D., Guan G., Xiao Y., Wang D. (2010). Multiblock copolymers composed of poly (butylene succinate) and poly (1, 2-propylene succinate): Effect of molar ratio of diisocyanate to polyester-diols on crosslink densities, thermal properties, mechanical properties and biodegradability. Polym. Degrad. Stab..

[32] Abdul Azam F.A., Rajendran Royan N.R., Yuhana N.Y., Mohd Radzuan N.A., Ahmad S., Sulong A.B. (2020). Fabrication of porous recycled HDPE biocomposites foam: Effect of rice husk filler contents and surface treatments on the mechanical properties. Polymers.

[33] Tangpasuthadol V., Intasiri A., Nuntivanich D., Niyompanich N., Kiatkamjornwong S. (2008). Silica-reinforced natural rubber prepared by the sol–gel process of ethoxysilanes in rubber latex. J. Appl. Polym. Sci..

[34] Kemaloglu S., Ozkoc G., Aytac A. (2010). Properties of thermally conductive micro and nano size boron nitride reinforced silicon rubber composites. Thermochim. Acta.

[35] Mohan T., Kuriakose J., Kanny K. (2011). Effect of nanoclay reinforcement on structure, thermal and mechanical properties of natural rubber–styrene butadine rubber (NR–SBR). J. Ind. Eng. Chem..

[36] Chong E., Ahmad I., Dahlan H., Abdullah I. (2010). Reinforcement of natural rubber/high density polyethylene blends with electron beam irradiated liquid natural rubber-coated rice husk. Radiat. Phys. Chem..

[37] Phomrak S., Phisalaphong M. (2020). Lactic acid modified natural rubber–bacterial cellulose composites. Appl. Sci..

[38] Samaržija-Jovanović S., Jovanovic V., Markovic G., Zeković I., Marinović-Cincović M. (2014). Properties of vulcanized polyisoprene rubber composites filled with opalized white tuff and precipitated silica. Sci. World J..

[39] Ismail H., Edyham M., Wirjosentono B. (2002). Bamboo fibre filled natural rubber composites: The effects of filler loading and bonding agent. Polym. Test..

[40] Karmarkar A., Chauhan S., Modak J.M., Chanda M. (2007). Mechanical properties of wood–fiber reinforced polypropylene composites: Effect of a novel compatibilizer with isocyanate functional group. Compos. Part A Appl. Sci. Manuf..

[41] Thomas M.G., Abraham E., Jyotishkumar P., Maria H.J., Pothen L.A., Thomas S. (2015). Nanocelluloses from jute fibers and their nanocomposites with natural rubber: Preparation and characterization. Int. J. Biol. Macromol..

[42] Timothy J.J., Meschke G. (2016). A cascade continuum micromechanics model for the effective elastic properties of porous materials. Int. J. Solids Struct..

[43] Nascimento R.M.D., Ramos S.M., Bechtold I.H., Hernandes A.N.C. (2018). Wettability study on natural rubber surfaces for applications as biomembranes. ACS Biomater. Sci. Eng..

[44] Wenzel R.N. (1936). Resistance of solid surfaces to wetting by water. Ind. Eng. Chem..

[45] Cassie A., Baxter S. (1944). Wettability of porous surfaces. Trans. Faraday Soc..

[46] Kirdponpattara S., Phisalaphong M., Newby B.-m.Z. (2013). Applicability of Washburn capillary rise for determining contact angles of powders/porous materials. J. Colloid Interface Sci..

[47] Frone A.N., Panaitescu D.M., Chiulan I., Nicolae C.A., Casarica A., Gabor A.R., Trusca R., Damian C.M., Purcar V., Alexandrescu E. (2018). Surface treatment of bacterial cellulose in mild, eco-friendly conditions. Coatings.

[48] Yang H., Yan R., Chen H., Lee D.H., Zheng C. (2007). Characteristics of hemicellulose, cellulose and lignin pyrolysis. Fuel.

[49] Mani T., Murugan P., Abedi J., Mahinpey N. (2010). Pyrolysis of wheat straw in a thermogravimetric analyzer: Effect of particle size and heating rate on devolatilization and estimation of global kinetics. Chem. Eng. Res. Des..

[50] Wang Z., McDonald A.G., Westerhof R.J., Kersten S.R., Cuba-Torres C.M., Ha S., Pecha B., Garcia-Perez M. (2013). Effect of cellulose crystallinity on the formation of a liquid intermediate and on product distribution during pyrolysis. J. Anal. Appl. Pyrolysis.

[51] Pichayakorn W., Suksaeree J., Boonme P., Taweepreda W., Ritthidej G.C. (2012). Preparation of deproteinized natural rubber latex and properties of films formed by itself and several adhesive polymer blends. Ind. Eng. Chem. Res..

[52] Farhadinejad Z., Ehsani M., Khosravian B., Ebrahimi G. (2012). Study of thermal properties of wood plastic composite reinforced with cellulose micro fibril and nano inorganic fiber filler. Eur. J. Wood Wood Prod..

